# Invasive Fungal Rhinosinusitis with and without Orbital Complications: Clinical and Laboratory Differences

**DOI:** 10.3390/jof7070573

**Published:** 2021-07-18

**Authors:** Kuan-Hsiang Twu, Ying-Ju Kuo, Ching-Yin Ho, Edward C. Kuan, Wei-Hsin Wang, Ming-Ying Lan

**Affiliations:** 1 Department of Otolaryngology-Head and Neck Surgery, Taipei Veterans General Hospital, Taipei 11217, Taiwan; wingstwu@gmail.com; 2 School of Medicine, National Yang Ming Chiao Tung University, Taipei 11221, Taiwan; yjkuo2@vghtpe.gov.tw (Y.-J.K.); tigertpe@gmail.com (C.-Y.H.); weihsin0103@gmail.com (W.-H.W.); 3 Department of Pathology, Taipei Veterans General Hospital, Taipei 11217, Taiwan; 4 Department of Otolaryngology, Cheng Hsin General Hospital, Taipei 11220, Taiwan; 5 Department of Otolaryngology-Head and Neck Surgery, University of California, Irvine, CA 92868, USA; eckuan@hs.uci.edu; 6 Department of Neurosurgery, Taipei Veterans General Hospital, Taipei 11217, Taiwan

**Keywords:** invasive fungal rhinosinusitis, orbital complications, fungal rhinosinusitis, *Aspergillus*, Mucor

## Abstract

Background: Invasive fungal rhinosinusitis (IFS) is a rare but often fatal disease. There are limited studies regarding IFS with orbital complications (IFSwOC). The present study aimed to identify the clinical signs associated with IFSwOC and prognosticators of the disease. Methods: A retrospective case series was conducted of patients histopathologically confirmed IFS or fungal rhinosinusitis with clinically apparent neuro-orbital complications who underwent surgery between 2008 and 2018. Demographic data, presenting symptoms and signs, culture data, laboratory results, and patient outcomes were obtained from medical records. Results: A total of 38 patients were identified, including 9 patients with IFSwOC, and 29 patients with IFS without orbital complications (IFSsOC). The clinical signs associated with developing orbital complications include headache, fever, sphenoid sinus, or posterior ethmoid sinus involvement, CRP level ≥ 1.025 mg/dL, or ESR level ≥ 46.5 mm/h. In IFSwOC group, male, posterior ethmoid sinus involvement, WBC count ≥ 9000 μL, CRP level ≥ 6.91 mg/dL, or ESR level ≥ 69 mm/h were correlated with a significantly poorer prognosis. Conclusion: IFS patients with sphenoid or posterior ethmoid sinus involvement, headache or fever as presenting symptoms, elevated CRP, and ESR level were at risk of developing orbital complications. Timely surgical debridement followed by systemic antifungal treatment may improve treatment outcomes.

## 1. Introduction

The diagnosis of invasive fungal rhinosinusitis (IFS) is made by histopathologic confirmation of fungal hyphae invading mucosal or submucosal tissues, vasculature, or bones [1]. According to differences in histopathology, IFS may be classified into three categories: acute invasive fungal rhinosinusitis (AIFS), chronic invasive fungal rhinosinusitis (CIFS), and granulomatous invasive fungal rhinosinusitis (GIFS) [1]. Although rare, IFS is a challenging condition for otolaryngologists owing to its tendency to cause serious injury to surrounding vital structures and association with high mortality rates if left untreated. According to a large systemic review of 807 patients of AIFS, 49.6% developed orbital invasion, 21.2% had intracranial extension, and 8.6% had cavernous sinus involvement [2]. Based on previous studies, reported mortality rate of AIFS is usually over 50% and can be as high as 80% [2,3,4,5].

Among the various complications of IFS, almost half of patients develop orbital complications [2], including orbital cellulitis, orbital abscess, decreased vision, proptosis, and diplopia. It is known that intracranial extension negatively impacts survival of patients with IFS [2,6,7,8,9]. Limited reports have found that orbital involvement was not a negative prognostic factor in IFS [2], and orbital exenteration did not appear to improve survival [2,10].

Many fungal species have been found to cause IFS, but the most commonly identified organisms are Aspergillus and Mucor (Zygomycetes) [2,3,5,11]. After fungus breaks through sinonasal mucosa, it can spread to the orbital apex, cavernous sinus, and finally intracranial structures. Once angioinvasion occurs, thrombosis and mycotic aneurysms could develop, resulting in ischemia, infarction, intracranial hemorrhage, and other neurologic symptoms [12,13,14,15,16]. Whether different fungal species had an impact on survival is still controversial. There is evidence suggesting that IFS due to mucormycosis was associated with a higher mortality rate than those related to Aspergillus infections [5,17]. On the other hand, a systematic review conducted by Turner et al. showed no statistically significant impact on overall survival among different types of fungal species [2].

Because of the rarity of IFS, there are few studies focusing on orbital complications in IFS, and are generally limited to case series. The objective of the current study is to identify the clinical signs associated with IFSwOC and also to seek possible factors that could negatively impact treatment outcomes of IFS with orbital involvement.

## 2. Materials and Methods

### 2.1. Patients

All procedures performed in studies involving human participants were in accordance with the ethical standards of the Institutional Review Board (IRB) of Taipei Veterans General Hospital (IRB NO: 2018-12-004CC), and with the 1964 Helsinki declaration and its later amendments or comparable ethical standards. We retrospectively reviewed the medical records of patients who had histopathologically confirmed IFS or fungal rhinosinusitis with clinically apparent neuro-orbital complications who underwent surgery between November 2008 and May 2018 at a tertiary academic medical center. Neuro-orbital complications included diplopia, blurred vision, proptosis, ptosis, or other central neurological signs/symptoms, such as hemiparesis, seizure, or altered mental status. Patients less than 18 years of age, or those who did not receive surgical treatment, were excluded.

Demographic data, underlying medical problems, presenting symptoms and signs, fungal and bacterial culture results, laboratory results, imaging studies, use of antifungal agents, and survival outcomes were obtained. The patients were divided into two groups based on whether they had orbital complications: IFS with orbital complications (IFSwOC) and IFS without orbital complications (IFSsOC). In the orbital complication group, the endpoint was defined as either mortality or long-term unrecovered sequelae.

### 2.2. Statistical Analysis

Chi-square tests or Fisher’s exact tests were used for comparisons of categorical variables. Independent Student’s t-tests or Mann–Whitney U tests were used for comparisons of continuous variables based on the distribution of the data. Receiver operating characteristic (ROC) curves and area under the curve (AUC) were used to analyze continuous variables, and cut-off values were calculated corresponding to the maximal value of Youden’s index. All statistical analyses were conducted using SPSS Statistics for Windows, Version 25.0 (Armonk, NY, USA: IBM Corp.). A *p*-value < 0.05 was considered statistically significant.

## 3. Results

### 3.1. Demographic Data, Lab Data, and Symptoms

**Figure 1:**
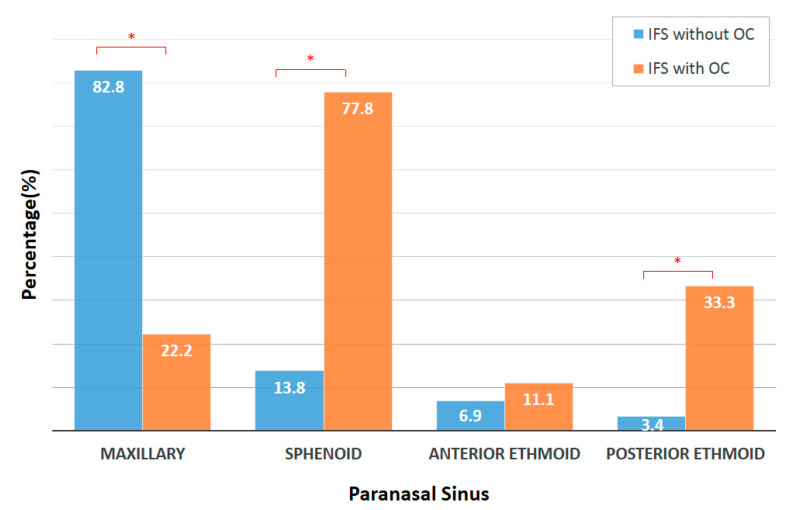
Sinus involvement in IFS. (* statistically significant (*p* < 0.05)).

Thirty-eight patients were included in this study. The mean age was 61.2 years (range 39–87 years); 24 were female and 14 were male. 9 (23.7%) patients presented with orbital complications. Six patients (15.8%) had AIFS and 32 patients (84.2%) had CIFS. Table 1 demonstrates baseline patient demographic data. There was no significant difference between the two groups regarding age, gender, laterality of involvement, and underlying comorbidities (*p* > 0.05). None of the patients in either group was neutropenic at the time of diagnosis; in addition, IFSwOC group had a higher absolute neutrophil count (10,229.56 cells/mm^3^) than IFSsOC group (4194.62 cells/mm^3^), although it was not statistically significant (*p* > 0.05). When comparing the involved sinuses between the two groups, it was found that sphenoid and posterior ethmoid sinus involvement was significantly more prevalent in the IFSwOC group (*p* = 0.035 and 0.001, respectively), whereas maxillary sinus involvement was more common in IFSsOC (*p* = 0.002) (Figure 1). Anterior ethmoid sinus involvement showed no significant difference between the two groups, and there were no cases presented with frontal sinus disease in our cohort. In the IFSwOC group, the common initial presentations included headache (*n* = 7, 77.8%), diplopia (*n* = 6, 66.7%), and fever (*n* = 5, 55.6%) (Table 1). In the IFSsOC group, the most common presenting symptom was purulent rhinorrhea (*n* = 14, 48.3%), followed by nasal obstruction (*n* = 8, 27.6%). In addition to orbital symptoms, IFSwOC group had statistically significantly higher rates of headache and fever (*p* = 0.002 and <0.001, respectively) than IFSsOC group. All of the patients got either a computed tomography (CT), magnetic resonance imaging (MRI), or both, at the time of diagnosis. Seven patients in IFSwOC group could identify cavernous sinus and/or orbital involvements on the scans, and no patients in IFSsOC group had positive findings for cavernous sinus or orbital invasion.

### 3.2. Microbiology

**Figure 2:**
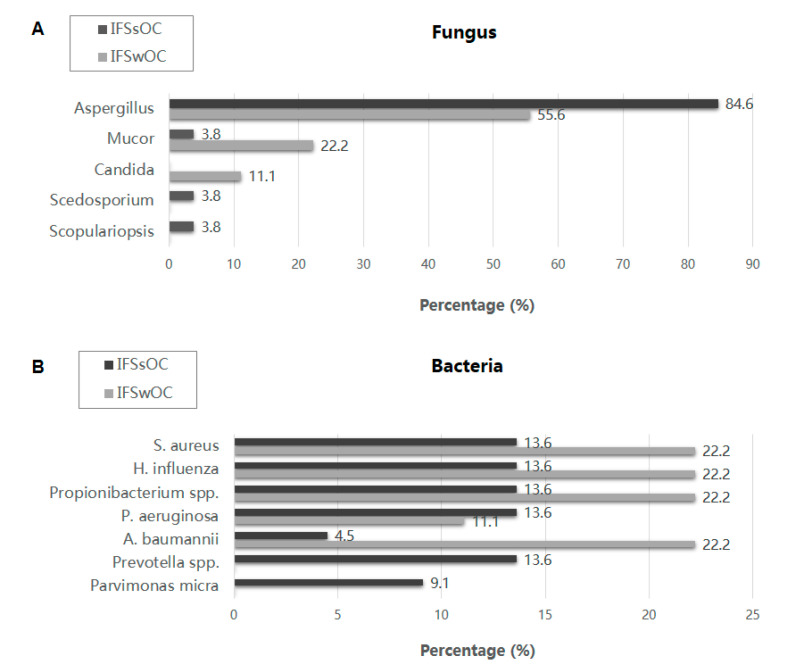
Microbiology in IFS (**A**) fungus and (**B**) bacteria.

Overall, Aspergillus was the predominant identified fungal species (77.1%), followed by Mucor (8.6%) and Scedosporium (2.9%) (Supplementary Table S1) (Figure 2). Fungal rhinosinusitis is usually accompanied with bacteria co-infection. Among the various isolated bacteria species, Hemophilus influenza, Staphylococcus aureus, and Propionibacterium spp. were most commonly seen (16.1% each). There was no statistically significant difference in the prevalence of causative fungal or bacterial pathogens between the two groups. The mean white blood cell (WBC) count was higher in the IFSwOC group (13,300 cells/mm^3^) than in the IFSsOC group (6686 cells/mm^3^), though this did not reach statistical significance (*p* = 0.066). In the IFSwOC group, the mean C-reactive protein (CRP) and erythrocyte sedimentation rate (ESR) level was 9.79 mg/dL and 62.88 mm/h, respectively, whereas the mean CRP and ESR level in IFSsOC group was 0.53 mg/dL and 28.67 mm/h, respectively. Both CRP and ESR level were significantly higher in IFSwOC group than IFSsOC group (*p* < 0.001 and *p* = 0.031, respectively).

### 3.3. Treatment and Outcome

We investigated treatment modalities and associated outcomes (Table 2). In addition to surgical treatment, all (100%) patients with orbital complications received systemic antifungal therapy, while only 65.5% of IFSsOC patients had systemic antifungal treatment. In accordance with microbiology, the most commonly used antifungal agent was voriconazole. Mean follow-up duration was 275 days (range 13 to 1303 days). Overall, three out of nine patients in IFSwOC group had poor prognoses (Supplementary Table S2). On the other hand, all patients in IFSsOC group improved clinically without any complications. Mortality rate was 2.6% in all patients, 11.1% in IFSwOC group, and 16.7% in AIFS patients.

### 3.4. Clinical Signs Associated with IFSwOC

**Figure 3:**
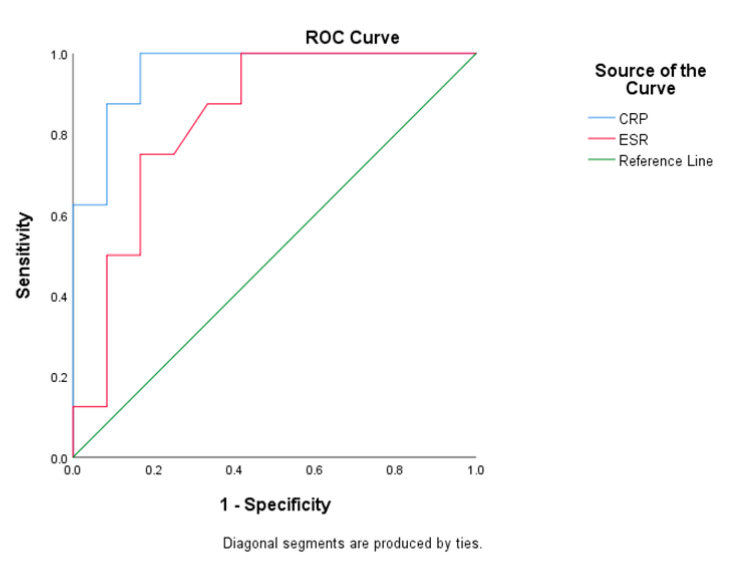
ROC graph for CRP and ESR. ROC curve of CRP and ESR as a risk factor of developing orbital complications in patients with IFS. The cutoff value of CRP was 1.025 mg/dL with a sensitivity of 0.875, a specificity of 0.917, and AUC of 0.958 (*p* = 0.001). The cutoff value of ESR was 46.5 mm/h with a sensitivity of 0.750, a specificity of 0.833, and AUC of 0.839 (*p* = 0.012).

Table 3 summarizes the clinical signs associated with IFSwOC. IFS patients have a statistically significantly higher risk for developing orbital complications if they had headache (OR 16.8, 95% CI 2.66 to 106.14, *p* = 0.002) or fever (*p* < 0.001), sphenoid (OR 21.88, 95% CI 3.295 to 145.28, *p* = 0.001), or posterior ethmoid sinus (OR 14.0, 95% CI 1.23 to 158.84, *p* = 0.035) involvement, CRP level ≥ 1.025 mg/dL (OR 88.0, 95% CI 4.76 to 1627.7, *p* < 0.001) or ESR level ≥ 46.5 mm/h (OR 15.0, 95% CI 1.65 to 136.17, *p* = 0.019). Figure 3 demonstrated ROC analysis. The cutoff value of CRP was 1.025 mg/dL with a sensitivity of 0.875, a specificity of 0.917, and AUC of 0.958 (*p* = 0.001). The cutoff value of ESR was 46.5 mm/h with a sensitivity of 0.750, a specificity of 0.833, and AUC of 0.839 (*p* = 0.012). Of the nine patients in IFSwOC group, three had poor prognosis, including one mortality and two with permanent neurologic sequelae (Supplementary Table S2). We compared these three patients with the other six patients in IFSwOC group who initially had orbital complications but fully recovered from the event. IFSwOC patients who were male, or who had posterior ethmoid sinus involvement, WBC count ≥ 9000 μL, CRP level ≥ 6.91 mg/dL, or ESR level ≥ 69 mm/h, had a significantly poorer prognosis (Supplementary Table S3).

## 4. Discussion

Our study is the first one to identify the clinical signs associated with IFSwOC and its prognosticators. From our results, we found that IFS patients were at risk of developing orbital complications if they had the risk factors, including sphenoid and posterior ethmoid sinus involvement, headache and fever as presenting symptoms, and elevated CRP (≥1.025 mg/dL) and ESR (≥46.5 mm/h) level.

In this study, the IFSwOC group more frequently had posterior ethmoid and sphenoid sinus involvement than the IFSsOC group. Furthermore, IFSwOC patients with posterior ethmoid sinus involvement had significantly worse survival outcomes. In IFS patients with neuro-orbital complications, it is believed that the fungal invasion usually originates from the paranasal sinuses and progresses through the mucosa and into the vasculature. After invading through the submucosal tissue, bone, and adjacent structures, fungi could directly invade the orbit, and then spread to the cavernous sinus and intracranial structures, causing serious complications [12]. The posterior ethmoid and sphenoid sinuses border the medial orbit, with only the thin lamina papyracea in between. There is minimal physical resistance for fungus to break through the thin bone and invade the orbit. On the other hand, the roof of the maxillary sinus (orbital floor) is much thicker than the lamina papyracea. This could explain why IFS in the maxillary sinus alone less commonly causes orbital symptoms. Similarly, the cavernous sinus and optic nerve are located at the posterolateral aspect of the sphenoid sinus, and they may be compromised in a similar manner. Direct fungal invasion into the cavernous sinus results in cavernous sinus thrombosis, which is a disastrous condition that would lead to orbital symptoms such as ptosis, chemosis, ophthalmoplegia, vision impairment, and blindness. Headache, fever, and sepsis could also develop, with the possibility of mortality. One of our patients suffered bilateral cavernous sinus thrombosis, and histopathology of the medial wall of cavernous sinus confirmed invasion by fungal hyphae.

In the present study, we attempted to identify risk factors of IFS leading to orbital complications. We found that the IFSwOC group had significantly more patients presenting with headache and fever, with a prevalence of 77.8% and 55.6%, respectively, compared with the IFSsOC group. Turner et al. conducted a systematic review of 807 IFS patients, showing that 62.9% and 46.3% of patients had fever and headache, respectively, as presenting symptoms, but they did not compare the prevalence between subgroups [2]. In fact, to the best of our knowledge, the current study is the first study to compare factors between IFSwOC and IFSsOC groups. Our results may suggest that, if IFS patients presented with either fever or headache, clinicians should be aware of the possibility of developing orbital complications.

According to previous reports, elevated CRP level and severe neutropenia are significantly correlated with worse survival [5,11]. Gode et al. reported that CRP level > 4 mg/dL was associated with poor prognosis in AIFS patients [5]. Cho et al. also suggested that patients with a CRP level > 5.5 mg/dL had worse outcomes [11]. In this study, we found that elevated CRP ≥ 1.025 mg/dL significantly increased the risk of developing orbital complications in IFS patients, and a CRP level ≥ 6.91 mg/dL was significantly associated with poorer prognosis in IFSwOC patients. The cut-off values were calculated corresponding to the maximal value of Youden’s index. Our findings were consistent with prior studies suggesting that elevated CRP level was a strong determinant of negative outcome. In addition, our series also discovered that WBC and ESR also were factors significantly related to IFSwOC. Specifically, ESR ≥ 46.6 mm/h predicted orbital involvement in IFS patients. In the IFSwOC group, WBC ≥ 9000/μL and ESR ≥ 69 mm/h both indicated poor prognosis. These results suggest that, if localized fungal rhinosinusitis progressed to systemic inflammation, the risks of permanent morbidities appeared to be higher.

In our series, nine of 38 (23.7%) IFS patients presented with orbital symptoms. The prevalence of orbital disease in our study is lower than in prior studies. Turner et al. reported that orbital involvement was the most commonly seen complications of IFS, with a 49.6% prevalence rate in AIFS patients [2]. Chandrasekharan et al. found out that 73.5% of patients with IFS had orbital involvement, while only 12.1% of patients with non-invasive fungal rhinosinusitis had orbit compromise [18]. Trief et al. discovered that about 58.3% of patients with IFS had orbital disease, and those with orbital involvement, had higher mortality rates comparing with orbit sparing cases [19]. Our lower prevalence of orbital disease in our study may be due to the majority of our patients were CIFS, accounting for 84.2% of total patients, while AIFS was only 15.8%. If we look at AIFS cases alone, six of six (100%) patients had orbital involvement, while in CIFS patients only 9.4% had orbital disease. CIFS is known to have a more indolent disease course than AIFS, and it is possible that most of the CIFS patients had been diagnosed with the disease before orbital involvement occurred. 

AIFS more commonly develops in immunocompromised patients, such as those with uncontrolled diabetes mellitus, severe neutropenia, or hematologic malignancies, those who are undergoing chemotherapy, or patients receiving bone marrow transplantation [2,11,20]. CIFS, on the contrary, occurs more frequently in immunocompetent hosts [21,22]. Our series revealed that, in 38 patients with IFS, none of them had hematologic disease, corticosteroid use, neutropenic status, or was receiving chemotherapy, and only 18.4% patients had diabetes mellitus. Even with subgroup analysis, only 33% of patients in the IFSwOC group had diabetes mellitus. In addition, there was no statistically significant difference between the IFSwOC and IFSsOC groups regarding their underlying medical comorbidities. Again, this may be accounted for the fact that the majority of cases had CIFS, which tend to consist of immunocompetent patients. However, the six patients with AIFS also had no underlying immunocompromised status. One possibility for this is small sample size. There are only few cases of IFS on immunocompetent hosts published in the previous literatures [23]. Though less prevalent, immunocompetent patients may develop AIFS as well, and they may also be at risk of suffering long-term complications, as two of our patients developed permanent neurologic sequelae and another one died of intracranial hemorrhage.

Fungal rhinosinusitis has been frequently found to be accompanied by bacteria co-infection. In our study, Aspergillus was the predominant identified fungal species, followed by Mucor, while Hemophilus influenza and Staphylococcus aureus were the most common isolated bacteria species. Superantigens and exotoxins of Staphylococcus aureus have been proved to participate in biofilm formation as well as impairment of local host immunity, which could contribute to the persistence and invasion of Staphylococcus aureus [24]. Similarly, various virulence factors of Hemophilus influenza involved in complement resistance, biofilm formation, and evasion of the host immune response promote the adhesion and internalization of Hemophilus influenza [25]. Therefore, mucosa barrier dysfunction and disrupted local immune system caused by certain bacterial infections, such as Staphylococcus aureus and Hemophilus influenza, may facilitate the invasion of fungus, contributing to the pathogenesis of IFS in both immunocompromised and immunocompetent patients. There are very few studies focusing on microbiota in fungal rhinosinusitis and fungal microbiota in chronic rhinosinusitis [26,27,28]. Our recent study using Next Generation Sequencing (NGS) technology uncovered the different bacterial and fungal microbiota between invasive and noninvasive fungal rhinosinusitis (unpublished data). We proposed that dysbiosis of sinus microbiota may play important role in the pathogenesis of IFS. Further investigation of fungal and bacterial microbiota interaction in IFS is needed in the future.

The mainstay of treatment for IFS is combining surgical debridement with systemic anti-fungal agents. Systemic anti-fungal therapies are an essential part of managing IFS. Amphotericin and the liposomal version are proved to have positive predictive value to survival [2,29]. Voriconazole is recommended for aspergillosis, and newer Azoles, such as isavuconazole and posaconazole, showing better bioavailability and less toxicity, are especially suitable for patients who have kidney disease or are intolerant to amphotericin B [29,30,31,32]. Combination therapy with two drugs (liposomal amphotericin B and isavuconazole or posaconazole) have been reported in cases with rhino–orbital–cerebral mucormycosis [32,33]. Surgical intervention significantly improves clinical outcomes and survival for patients with IFS [2,9,34], and endoscopic surgery has been reported to yield successful and comparable treatment outcomes with open approaches [2,35,36].

According to prior works, reported mortality rates for IFS ranged from 17.8% to 85.7% [5,11,12,17,35,37,38,39,40]. In the present cohort, overall mortality rate was 2.6% in all patients, including 11.1% in the IFSwOC group and 16.7% among AIFS patients. Comparing only AIFS with cases from prior studies, the current study’s mortality rate is lower, which could be credited to the following factors. First, all patients underwent surgical debridement, and all patients with orbital complications received systemic anti-fungal treatment. Even in the IFSsOC group, more than 65% of patients received postoperative systemic anti-fungal medications. This underscores the importance of aggressive debridement with antifungal therapy in managing this disease optimally. Second, there could be selection bias in our study population, as all of them enrolled in this study were patients deemed appropriate for surgical treatment. Those inoperable patients with clinically suspicious fungal rhinosinusitis were not included in the study owing to the lack of pathologic diagnosis. Consequently, the mortality rate could be underestimated, especially those who were inoperable often had poor general condition or high anesthetic risks.

Part of the key of successful surgical treatment is to debride as much diseased/necrotic sinonasal tissue as possible in order to lower the fungal load to a minimum. In clinical practice, however, it is not always possible to sample involved tissues for diagnosis, especially if the area of invasion is deep or high-risk (e.g., skull base, carotid artery). The lack of histopathologic proof does not rule out an invasive process. The European Organization for Research and Treatment of Cancer (EORTC) and the Mycoses Study Group (MSG) in 2019 has revised and updated their latest version of the Consensus Definitions of Invasive Fungal Disease in 2019 [41]. They classified invasive fungal disease into proven, probable, and possible categories. Proven invasive fungal disease is defined as evidence of fungal elements in the specimen. Diagnosis of probable or possible invasive fungal sinonasal diseases does not necessarily require a histopathologic proof of invasion of fungi into tissue, but it is proposed for immunocompromised patients. Nevertheless, this criteria does not fit into IFS well, as IFS can occur in immunocompetent patients. Liang et al. observed sinonasal fungal infection demonstrating transitional changes between each classification [42], which corresponds to Kosmidis’s proposal that different forms of pulmonary aspergillosis should be viewed as a semicontinuous spectrum of disease and one form may evolve into another [43]. Therefore, we proposed that although there was no histopathologic evidence of invasive fungal disease, if the patient suffered a fungal rhinosinusitis with clinically apparent neuro-orbital complications, the patient still would benefit from aggressive treatment with early surgical debridement followed by systemic anti-fungal medications.

There are several limitations to the present study. This is a retrospective single institutional study in a tertiary referral center. Some data were missing or unclear in the medical records. The sample size is small, and it is even smaller with subgroup analysis, which decreases statistical power. As previously mentioned, there might be selection bias in our cohort, since inoperable patients were not included in the study. Further prospective studies with larger sample size are needed to establish the key risk factors and prognostic factors related to orbital complications in IFS patients.

## 5. Conclusions

To the best of our knowledge, this is the first study to identify the clinical signs associated with IFSwOC and prognosticators of the disease. Patients with invasive fungal rhinosinusitis who had sphenoid and posterior ethmoid sinus involvement, headache and fever as presenting symptoms, and elevated CRP (≥1.025 mg/dL) and ESR (≥46.5 mm/h) level were at risk of developing orbital complications. Timely surgical debridement followed by systemic anti-fungal treatment could yield satisfactory treatment outcomes.

## Figures and Tables

**Table 1:** Comparison of demographic data, lab data, symptoms and imaging results between IFSsOC and IFSwOC.

	All Patients (*n* = 38) No. (%)	IFSsOC (*n* = 29) No. (%)	IFSwOC (*n* = 9) No. (%)	*p* Value
**Age (mean ± SD)**	61.18 ± 12.30	60.03 ± 12.10	64.89 ± 12.96	0.308
**Gender (male: female)**	14:24	11:18	3:6	1.000
**Classification**				**<0.001**
Acute invasive	6 (15.8)	0 (0.0)	6 (66.7)	
Chronic invasive	32 (84.2)	29 (100.0)	3 (33.3)	
**Side**				
Left	22 (57.9)	17 (58.6)	5 (55.6)	1.000
Right	18 (47.4)	12 (41.4)	6 (66.7)	0.260
**Location**				
Maxillary	26 (68.4)	24 (82.8)	2 (22.2)	**0.002**
Sphenoid	11 (28.9)	4 (13.8)	7 (77.8)	**0.001**
Anterior ethmoid	3 (7.9)	2 (6.9)	1 (11.1)	1.000
Posterior ethmoid	4 (10.5)	1 (3.4)	3 (33.3)	**0.035**
Others *	2 (5.3)	2 (6.9)	0 (0.0)	1.000
**Underlying Diseases**				
Hypertension	11 (28.9)	6 (20.7)	5 (55.6)	0.088
Diabetes mellitus	7 (18.4)	4 (13.8)	3 (33.3)	0.322
Coronary artery disease	5 (13.2)	3 (10.3)	2 (22.2)	0.574
Chronic kidney disease	1 (2.6)	1 (3.4)	0 (0.0)	1.000
Hyperlipidemia	5 (13.2)	4 (13.8)	1 (11.1)	1.000
Atrial fibrillation	2 (5.3)	1 (3.4)	1 (11.1)	0.422
Autoimmune disease	2 (5.3)	2 (6.9)	0 (0.0)	1.000
**Lab Data**				
WBC (/μL)	8252.63 ± 5850.74	6686.21 ± 1796.97	13,300.00 ± 10,462.43	0.066
ANC (/μL)	5623.95 ± 5660.50	4194.62 ± 1619.37	10,229.56 ± 10,379.96	0.103
CRP (mg/dL)	4.50 ± 8.17	0.53 ± 0.92	9.79 ± 10.52	**<0.001**
ESR (mm/h)	42.35 ± 32.37	28.67 ± 23.34	62.88 ± 34.44	**0.031**
**Symptoms Duration** **(days, mean ± SD)**	236.22 ± 631.58	297.52 ± 736.53	79.56 ± 124.36	0.389
Purulent rhinorrhea	14 (36.8)	14 (48.3)	0 (0.0)	**0.014**
Headache	12 (31.6)	5 (17.2)	7 (77.8)	**0.002**
Nasal obstruction	8 (21.1)	8 (27.6)	0 (0.0)	0.159
Diplopia	6 (15.8)	0 (0.0)	6 (66.7)	**<0.001**
Fever	5 (13.2)	0 (0.0)	5 (55.6)	**<0.001**
Foul odor	5 (13.2)	5 (17.2)	0 (0.0)	0.312
Blood-tinged rhinorrhea	4 (10.5)	4 (13.8)	0 (0.0)	0.554
Facial pain/fullness	4 (10.5)	4 (13.8)	0 (0.0)	0.554
Blurred vision	3 (7.9)	0 (0.0)	3 (33.3)	**0.010**
Proptosis	2 (5.3)	0 (0.0)	2 (22.2)	0.051
Ptosis	2 (5.3)	0 (0.0)	2 (22.2)	0.051
Facial numbness	2 (5.3)	2 (6.9)	0 (0.0)	1.000
Facial swelling	2 (5.3)	1 (3.4)	1 (11.1)	0.422
Consciousness disturbance	1 (2.6)	0 (0.0)	1 (11.1)	0.237
**Imaging Results**				
Cavernous sinus or orbital involvement suspected on CT/MRI	7 (18.4)	0 (0.0)	7 (77.8)	**<0.001**

Abbreviations: IFS, invasive fungal rhinosinusitis; SD, standard deviation; WBC, white blood cell; ANC, absolute neutrophil count; CRP, C-reactive protein; ESR, erythrocyte sedimentation rate; CT, computed tomography; MRI, magnetic resonance imaging. * other locations: 1 in nasal septum, 1 in bilateral maxillary sinuses, nasopharynx, clivus, and parapharyngeal space. Bold indicates statistically significant (*p* < 0.05).

**Table 2:** Treatment and Outcome.

	All Patients (*n* = 38) No. (%)	IFSsOC (*n* = 29) No. (%)	IFSwOC (*n* = 9) No. (%)	*p* Value
**Follow-up Duration (days)**	275.43 ± 262.01	286.76 ± 284.83	220.67 ± 90.26	0.983
**Surgery**				
Interval between onset of symptoms and surgery (days)	107.13 ± 99.19	145.14 ± 93.77	14.22 ± 11.38	**<0.001**
**Antifungal Therapy**				
Voriconazole	24 (63.2)	17 (58.6)	7 (77.8)	0.438
Amphotericin B	1 (2.6)	0 (0.0)	1 (11.1)	0.237
Liposomal amphotericin B	2 (5.3)	0 (0.0)	2 (22.2)	0.051
Others *	4 (10.5)	2 (6.9)	2 (22.2)	0.233
Duration (days)	73.85 ± 67.86	70.11 ± 79.32	82.25 ± 32.41	0.140
**Antibiotic**				
Duration (days)	27.71 ± 39.76	22.95 ± 43.83	42.00 ± 19.82	**0.005**
**Sequelae**	2 (5.3)	0 (0.0)	2 (22.2)	0.051
**Mortality**	1 (2.6)	0 (0.0)	1 (11.1)	0.237
**Sequelae or Mortality**	3 (7.9)	0 (0.0)	3 (33.3)	**0.010**

Abbreviations: IFS, invasive fungal rhinosinusitis; SD, standard deviation. Bold indicates statistically significant (*p* < 0.05). * Others included posaconazole and itraconazole.

**Table 3:** The clinical signs associated with IFSwOC.

	Odds Ratio	95% CI	*p* Value
**Location**			
Maxillary	0.060	0.009–0.376	0.002
Sphenoid	21.875	3.295–145.237	0.001
Posterior ethmoid	14.000	1.234–158.844	0.035
**Symptoms**			
Headache	16.800	2.659–106.135	0.002
Fever	8.25 *		<0.001
**Lab Data**			
CRP (≥1.025 mg/dL)	88.000	4.758–1627.704	<0.001
ESR (≥46.5 mm/h)	15.000	1.652–136.172	0.019

Abbreviations: IFS, invasive fungal rhinosinusitis; CI, confidence interval; CRP, C-reactive protein; ESR, erythrocyte sedimentation rate; RR, relative risk ratio. * There is no case having fever in IFSsOC, hence having infinite odds ratio. RR was shown instead of odds ratio here.

## Supplementary Materials

The following are available online at https://www.mdpi.com/article/10.3390/jof7070573/s1, Table S1: Microbiology, Table S2: Clinical characteristics of 3 IFSwOC patients with poor prognosis, Table S3: Prognostic factors of IFSwOC.
