# Supplementary material for: Invasive Fungal Rhinosinusitis with and without Orbital Complications: Clinical and Laboratory Differences
Source: J Fungi (Basel). 2021 Jul 18;7(7):573. doi: 10.3390/jof7070573 (PMC8306043; doi:10.3390/jof7070573)
Supplement: Supplementary file 1 [file jof-07-00573-s001.zip › jof-1286695-supplementary.pdf]

Supplementary Data

Supplementary Table S1. Microbiology

	**All patients (n=35)**	**IFSsOC (n=26)**	**IFSwOC (n=9)**	***p* value**
**Fungus (%)**				
Aspergillus	27 (77.1%)	22 (84.6%)	5 (55.6%)	0.162
Mucor	3 (8.6%)	1 (3.8%)	2 (22.2%)	0.156
Scedosporium spp.	1 (2.9%)	1 (3.8%)	0 (0.0%)	1.000
Candida guilliermondii	1 (2.9%)	0 (0.0%)	1 (11.1%)	0.257
Scopulariopsis	1 (2.9%)	1 (3.8%)	0 (0.0%)	1.000
Not isolated	3 (8.6%)	2 (7.7%)	1 (11.1%)	1.000
	**All patients (n=31)**	**IFSsOC (n=22)**	**IFSwOC (n=9)**	***p* value**
**Bacteria (%)**				
Pseudomonas aeruginosa	4 (12.9%)	3 (13.6%)	1 (11.1%)	1.000
Hemophilus influenza	5 (16.1%)	3 (13.6%)	2 (22.2%)	0.613
Staphylococcus aureus	5 (16.1%)	3 (13.6%)	2 (22.2%)	0.613
Propionibacterium spp.	5 (16.1%)	3 (13.6%)	2 (22.2%)	0.613
Prevotella spp.	3 (9.7%)	3 (13.6%)	0 (0.0%)	0.537
Parvimonas micra	2 (6.5%)	2 (9.1%)	0 (0.0%)	1.000
Acinetobacter baumannii	3 (9.7%)	1 (4.5%)	2 (22.2%)	0.195
Not isolated	4 (12.9%)	3 (13.6%)	1 (11.1%)	1.000

Abbreviations: IFS, invasive fungal rhinosinusitis; spp., species
Bold indicates statistically significant ($p < 0.05$)

Supplementary Table S2. Clinical characteristics of 3 IFSwOC patients with poor prognosis

Case NO.	Symptoms	Sinus	Fungus	Clinical course	Mortality or sequella
**1**	Left eye proptosis, erythema, fever	Left ethmoid and bilateral sphenoid sinus	Mucormycosis	Left ICA, MCA dissecting pseudoaneursym, SAH, ICH, IVH	Mortality
**2**	Headache, fever, diplopia and ptosis	Right posterior ethmoid and maxillary sinuses	Aspergillus	Bilateral cavernous sinus thrombosis, skull base osteomyelitis, fungal meningitis	Bilateral CN2, right CN3, 6 palsies
**3**	Blurred vision, proptosis, hemiplegia	Right sphenoid and posterior ethmoid sinuses	Mucormycosis	Sepsis , right cerebral, cerebellar and brainstem infarctions	Right eye decreased vision and permanent neurologic deficit

Abbreviations: ICA: internal carotid artery; MCA: middle cerebral artery; SAH: subarachnoid hemorrhage; ICH: intracerebral hemorrhage; IVH: intraventricular hemorrhage; CN: cranial nerve

Supplementary Table S3. Prognostic factors of IFSwOC

	With sequelae or mortality (%)	*p* value
**All patients**	33.3	
**Age**		0.948
**Gender**		
Male	100.0	**0.012**
Female	0.0	**0.012**
**Side**		
Left	20.0	0.524
Right	33.3	1.000
**Location**		
Maxillary	100.0	0.083
Sphenoid	14.3	0.083
Anterior ethmoid	100.0	0.333
Posterior ethmoid	100.0	**0.012**
**Underlying diseases**		
Hypertension	60.0	0.167
Diabetes mellitus	66.7	0.226
Coronary artery disease	0.0	0.500
Hyperlipidemia	0.0	1.000
Atrial fibrillation	100.0	0.333
**Symptoms**		
Headache	14.3	0.083
Diplopia	16.7	0.226
Fever	40.0	1.000
Blurred vision	33.3	1.000
Facial swelling	100.0	0.333
Proptosis	100.0	0.083
Ptosis	50.0	1.000
Hemiparesis	100.0	0.333
Consciousness disturbance	100.0	0.333
Seizure	100.0	0.333
**Fungus**		
Aspergillus	20.0	0.524
Mucor	100.0	0.083
Candida guilliermondii	0.0	1.000

Not isolated	0.0	1.000
**Bacteria**		
Pseudomonas aeruginosa	100.0	0.333
Hemophilus influenza	50.0	1.000
Staphylococcus aureus	0.0	0.500
Propionibacterium spp.	0.0	0.500
Acinetobacter baumannii	100.0	0.083
**Lab data**		
WBC (≥ 9000μL)	75.0	**0.048**
CRP (≥ 6.91mg/dL)	75.0	**0.048**
ESR (≥ 69 mm/hour)	100.0	**0.036**
**Interval between onset of symptoms and surgery (≥ 10.5 days)**	50.0	0.524
**Antifungal therapy**		
Voriconazole	14.3	0.083
Amphotericin B	100.0	0.333
Liposomal amphotericin B	100.0	0.083
Others	100.0	0.083

Abbreviations: IFS, invasive fungal sinusitis; SD, standard deviation
Values in red indicate statistically significant ($p < 0.05$)
